# The association between declining lung function and stroke risk: insights from an observational study and Mendelian randomization

**DOI:** 10.3389/fneur.2024.1401959

**Published:** 2024-06-07

**Authors:** Jiadong Wang, Junjie Lin, Yujie Zheng, Minxia Hua, Kunyi Wang, Kexin Lu, Yu Zhang, Weijun Zheng, Rucheng Chen, Fuquan Lin

**Affiliations:** ^1^Hangzhou Third People’s Hospital, Hangzhou, China; ^2^Department of Clinical Medicine, School of Medicine, Hangzhou City University, Hangzhou, China; ^3^School of Public Health, Zhejiang Chinese Medical University, Hangzhou, China; ^4^Zhejiang International Science and Technology Cooperation Base of Air Pollution and Health, Hangzhou, China

**Keywords:** observational study, Mendelian randomization, stroke, peak expiratory flow, NHANES (National Health and Nutrition Examination Survey), CHARLS

## Abstract

**Background:**

Stroke, prevalent globally, particularly impacts low- and middle-income countries. Decreased lung function is one of the risk factors for stroke, and there is a lack of sufficient research on the association between the two, especially based on evidence from representative large samples. We aimed to explore the association between lung function and stroke incidence.

**Methods:**

We collected data from 13,371 participants from the 2007–2012 U.S. national cross-sectional study and 11,192 participants from the Chinese national cohort study during the 2011–2018 follow-up period. Multivariate logistic regression and Cox proportional hazards regression were used to assess cross-sectional and longitudinal associations of peak expiratory flow with stroke risks. Additionally, we used publicly available GWAS data from a European population to conduct Mendelian randomization analysis, further exploring the potential causal relationship.

**Results:**

The results of the cross-sectional study suggest that a decline in peak expiratory flow may be associated with an increased risk of stroke. The cohort study revealed that, compared to the first tertile group, the risk of stroke incidence in the second and third tertile groups of PEF decreased by 19% (hazard ratio (HR) = 0.810, 95%CI = 0.684–0.960) and 21.4% (HR = 0.786, 95%CI = 0.647–0.956), respectively. Mendelian randomization analysis clarified that higher PEF levels are significantly associated with a reduced risk of stroke (OR = 0.852, 95%CI = 0.727–0.997).

**Conclusion:**

Decreased lung function is a risk factor for stroke. As a simple and accurate indicator of lung function, PEF can be used to monitor lung function in community populations and patients for primary stroke prevention.

## 1 Introduction

Stroke is a prevalent cerebrovascular disease that manifests as a severe health issue due to impaired cerebral blood flow and is characterized by “five highs”: high incidence, high disability rate, high mortality rate, high recurrence rate, and high economic burden (1). Estimates indicate that approximately 7 million Americans aged >20 years self-report having experienced a stroke. Annually, there are approximately 795,000 new and recurrent stroke incidents in the United States. Analysis of the Global Burden of Disease Study data revealed that the overall prevalence of stroke is approximately 2.5%, ranking it as the second leading cause of death and the third leading cause of disability worldwide (1, 2). Although the incidence of stroke in developed countries has been steadily declining, stroke remains a major cause of death and long-term disability in low and middle-income countries (3). In 2019, approximately 3.94 million new stroke cases were recorded in China (4). Traditionally considered an age-related disease, stroke has shown an increasing incidence in the younger population in recent years, with a decrease in the age-standardized mortality rate but a continuous increase in the absolute number of patients, posing significant challenges to public health (2).

The physiological aging of the lungs and the decline in respiratory muscle strength, inevitable results of aging, lead to reductions in forced expiratory volume in one second (FEV1), forced vital capacity (FVC), and peak expiratory flow (PEF) (5). The PEF is a recognized method for measuring lung capacity, where readings depend on the patient’s voluntary effort and muscle strength. It is particularly suitable as an endpoint indicator for lung function testing in the home and community clinic settings and correlates well with the FEV1 measured by spirometry, effectively reflecting airway patency (6).

A decrease in lung function is closely related to impairments in both physical and psychological functions. Systematic reviews indicate that milder pulmonary diseases or subclinical lung function impairments may impact cardiovascular function and diseases (7). In particular, chronic obstructive pulmonary disease (COPD), characterized by chronic airflow limitation and an increased pulmonary inflammatory response, exhibits a bidirectional association with adverse cardiovascular events (8). However, the underlying mechanisms of this association remain unclear. Several potential mechanisms include pulmonary hyperinflation, hypoxemia, and pulmonary hypertension, all of which are markers of declining lung function (8, 9). Several studies utilizing community samples have investigated the association between a decrease in lung function and morbidity and mortality due to adverse cardiovascular events. A Mendelian randomization (MR) study that further subdivided cardiovascular diseases provided robust insights into the relationship between declining lung function and the risk of developing coronary artery disease (10). Previous research grouped various subtypes of cardiovascular diseases, including cerebrovascular diseases, together when investigating the relationship between lung function and cardiovascular health. This approach may overlook specific mechanisms and risk factors for each cardiovascular subtype, thereby limiting insights into how lung function affects these distinct conditions. A recent study used the UK Clinical Practice Research Datalink to explore the relationship between COPD and stroke (11).

Research on the association between declining lung function and stroke risk is limited. This study employs data from the National Health and Nutrition Examination Survey (NHANES), a comprehensive cross-sectional survey in the United States, and the Chinese Health and Retirement Longitudinal Survey (CHARLS), a nationwide cohort study in China, to investigate the relationship between declining lung function and stroke. To our knowledge, this research is the first to use extensive national epidemiological data for exploring this association. This study also provides inaugural evidence from a Chinese population, with additional MR analysis offering robust support for this association.

## 2 Materials and methods

### 2.1 Data source

**Figure 1:**
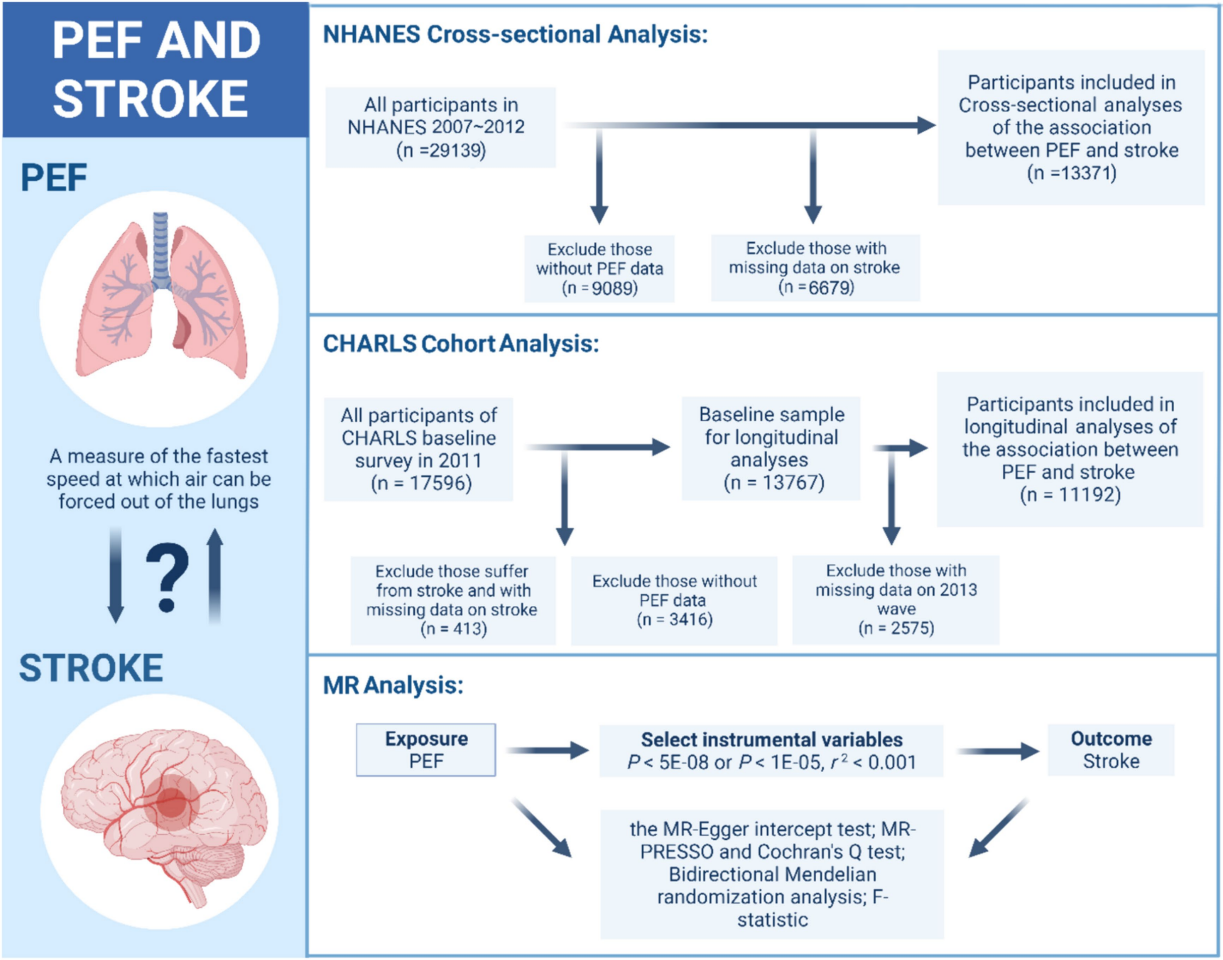
The flowchart for the observational study and MR analysis. PEF, peak expiratory flow; NHANES, National Health and Nutrition Examination Survey; CHARLS, Chinese Health and Retirement Longitudinal Survey; MR, Mendelian randomization.

The data for the observational study were derived from the 2007 to 2012 cycle of the NHANES and from the four waves of the CHARLS spanning from 2011 to 2018. The flowchart for all analyses is shown in Figure 1.

The NHANES, conducted annually by the National Center for Disease Control and Prevention, is a crucial tool in assessing the health and nutritional status of the U.S. population. This cross-sectional survey selects approximately 5,000 representative participants each year from various counties for detailed health and nutritional evaluations, combining in-person interviews with physical examinations in homes and mobile centers. A total of 29,139 subjects were enrolled in the 2007 to 2012 cycle. In this study, we excluded 9,089 participants due to missing PEF test data and 6,679 participants due to a lack of stroke data. Consequently, we included 13,371 participants to explore the cross-sectional association between PEF and stroke.

The CHARLS is a longitudinal survey conducted by the National School of Development at Peking University; it included individuals aged 45 years or older and their spouses throughout China. It assesses community residents’ social, economic, and health conditions by drawing representative samples from 28 provinces, 150 counties, and 450 communities, thereby ensuring its representativeness on a national scale. The interviewers utilized computer-aided personal interviewing systems during face-to-face surveys to accurately document participants’ responses and conducted follow-up examinations every 2 or 3 years (12). In this study, from the initial pool of 17,596 participants, 413 individuals were excluded due to either current stroke or incomplete stroke data. Furthermore, 3,416 participants who did not participate in the PEF test were also excluded from the analysis. Ultimately, we included 11,192 baseline study participants with seven-year follow-up data to analyze the longitudinal association between PEF and stroke. This study design follows the STROBE guideline, as detailed in Supplementary Table S1 (13).

For MR analysis, we utilized data from the UK Biobank and publicly available summarized Genome-Wide Association Study (GWAS) datasets. For the GWAS data on PEF, we employed the aggregated data from the UK Biobank. In this effort, the UK Biobank measured the PEF of 307,638 participants (14). For the GWAS data on stroke, we utilized data from the most extensive recent stroke GWAS analysis. This analysis, led by Rainer Malik, included data from 17 studies (excluding the UK Biobank) involving a total of 446,696 participants of European descent, including 40,585 cases and 406,111 controls. These cases covered all stroke phenotypes, including ischemic and hemorrhagic strokes, to identify loci associated with stroke risk (15). Figure 1 shows the details.

### 2.2 Measurement of peak expiratory flow

Participants in the PEF measurement must be at least 6 years old and are tested while standing, using a standardized protocol. Individuals unable to stand are permitted to complete the test while seated. Adult participants were required to exhale for at least 6 s.

In the NHANES, each participant may undergo up to eight repeat tests to ensure accurate and consistent spirometry readings, with some subjects being tested both before and after taking bronchodilators. Subsequently, experts at the NIOSH Quality Control Center actively reviewed and validated the gathered PEF data. In this study, we used the PEF data collected before participants took bronchodilators to reflect their baseline PEF levels.

In the CHARLS, participants underwent three PEF measurements at 30 s intervals. In this analysis, we used the highest value from the three exhalation maneuvers, as described in previous studies (16, 17).

### 2.3 Measurement of stroke

In this study, we identified stroke among participants using the Medical Condition Questionnaire, which is applicable in both the CHARLS and NHANES. Participants were required to answer, “Has a doctor ever informed you that you have had a stroke?” Those who answered “Yes” were classified as having a history of stroke, whereas those who responded “No” were categorized as not having had a stroke.

### 2.4 Assessment of covariates

In this study, we controlled for potential confounding covariates, including demographic variables such as age, gender, body mass index (BMI), race, place of residence, marital status, and educational level. Additionally, health-related behaviors including smoking status and alcohol consumption, as well as medical conditions related to PEF and stroke, namely hypertension, hyperlipidemia, and cardiovascular disease, were also considered in our analysis. Supplementary Table S2 shows the details.

### 2.5 Statistical analysis

We conducted descriptive statistics on the baseline characteristics of the participants and provided the mean and standard deviation of PEF for different subgroups.

We divided the PEF into tertiles, using the first tertile as the control group, and evaluated the odds ratio (OR) and 95% confidence interval (CI) for the cross-sectional association between PEF and stroke by the logistic regression. Additionally, we employed the Cox proportional hazards model to assess the hazard ratio (HR) and 95% CI for the longitudinal association. We conducted the logistic regression and Cox proportional hazards with three models: Model 1 included adjustments for age, gender, BMI, race/place of residence, marital status, and educational level; Model 2 included health-related behavior (smoking and drinking status); and Model 3 included hypertension, hyperlipidemia, and cardiovascular disease. Additionally, we conducted trend tests using the median PEF of each tertile group. In this study, we conducted multiple imputations for missing values in covariates. Supplementary Table S3 shows details of the proportion of missing data and the multiple imputation. We also employed three-knot restricted cubic splines to explore the potential nonlinear association between PEF and stroke.

With respect to the GWAS data for PEF, we identified single nucleotide polymorphisms (SNPs) that were significantly associated with PEF (*p* < 5 × 10^−8) and were relatively independent (r^2 < 0.001 with a clustering window of 10 Mb). Subsequently, we calculated the *F*-statistics for these selected SNPs using the formula β^2/SE^2 to mitigate the impact of weak instrument bias on our results (18). Finally, we harmonized the exposure and outcome data and removed palindromic SNPs (18). We employed the MR approach, utilizing instrumental variables for statistical analysis. When these instrumental variables satisfy the three core assumptions of MR, this method can be used to assess the causal relationship between exposure and outcomes effectively while remaining immune to confounding biases (19). We adopted a two-sample MR approach to evaluate the potential causal relationship between PEF and stroke. The sources and selection of the instrumental variables have been elaborately described earlier in the text. Our primary analytical method was the inverse variance weighted (IVW) approach, which possesses the highest statistical power under the fulfillment of MR assumptions (18, 19).

Additionally, we performed two sensitivity analyses to ensure the robustness of our observational study results: first, we analyzed the data before multiple imputation of covariates; second, we further examined the impact of stroke for each standard deviation increase in PEF. In MR analysis, we conducted supplementary analyses using the weighted median method and the MR-Egger approach. For sensitivity analysis, we principally employed the MR-Egger intercept to test for pleiotropy, complemented by MR-PRESSO for additional pleiotropic analysis (20). We also utilized Cochran’s Q test to examine the heterogeneity of the results (18). Finally, bidirectional MR was used to exclude the influence of potential reverse causation, and leave-one-out analysis was employed to detect whether any specific SNP significantly impacted the results (21).

Data processing and analysis were performed using R version 4.2.1, along with the Storm Statistical Platform.^1^ A two-sided *p*-value≤0.05 was considered statistically significant.

## 3 Results

### 3.1 Baseline characteristics

In the cross-sectional analysis of the NHANES, we included 13,371 participants with an average age of 46.68 ± 16.27 years; 49.88% (6670) were males and 2.26% (302) stroke sufferers. For the cohort analysis, our sample consisted of 11,192 participants, aged 58.74 ± 9.49 years, 46.84% (5,240) of whom were male. Across both study types, we observed significant variations in PEF across sex, BMI, region, race, marital and educational status, drinking behavior, and conditions such as hypertension, hyperlipidemia, and heart disease. However, differences in PEF among individuals with different smoking statuses were detected only in the cohort study. Table 1 shows the details.

**Table 1:** Baseline participants and PEF characteristics of NHANES and CHARLS.

Variables	NHANES (*n* = 13,371)			CHARLS (*n* = 11,192)		
	Respondents^a^	PEF L/min (Mean ± SD)	*p* ^b^	Respondents^a^	PEF L/min (Mean ± SD)	*p* ^b^
**Total**	13,371	477.19 ± 135.15		11,192	299.11 ± 120.99	
**Age**	46.68 ± 16.27			58.74 ± 9.49		
**Gender**			<0.001			<0.001
Male	6,670 (49.88%)	553.08 ± 127.97		5,240 (46.84%)	348.59 ± 130.45	
Female	6,701 (50.12%)	401.66 ± 93.32		5,947 (53.16%)	255.52 ± 92.17	
**BMI**						<0.001
Normal weight	3,699 (27.80%)	476.63 ± 128.73		5,837 (52.96%)	298.84 ± 120.80	
Underweight	186 (1.40%)	406.71 ± 120.65		712 (6.46%)	242.71 ± 108.33	
Overweight/Obese	9,421 (70.80%)	479.25 ± 137.37		4,472 (40.58%)	310.03 ± 120.84	
**Race**			<0.001			/
Mexican American	2,117 (15.83%)	480.45 ± 132.10		/	/	
Other Hispanic	1,437 (10.75%)	465.18 ± 130.87		/	/	
Non-Hispanic White	5,705 (42.67%)	488.66 ± 136.31		/	/	
Non-Hispanic Black	2,930 (21.91%)	459.25 ± 135.20		/	/	
Other Race	1,182 (8.84%)	475.11 ± 134.11		/	/	
**Place of residence**			/			0.007
Urban	/	/		7,256 (64.83%)	296.82 ± 120.62	
Rural	/	/		3,936 (35.17%)	303.33 ± 121.57	
**Marriage status**			<0.001			<0.001
Married/Living with a partner	8,044 (60.20%)	486.13 ± 131.81		9,885 (88.32%)	305.93 ± 120.92	
Widowed/Divorce/Separated	2,630 (19.68%)	420.98 ± 132.32		1,227 (10.96%)	244.84 ± 108.45	
Never married	2,687 (20.12%)	505.45 ± 132.52		80 (0.72%)	288.91 ± 100.48	
**Education level**			<0.001			<0.001
High school or below	6,436 (48.17%)	456.24 ± 136.72		11,019 (98.48%)	297.78 ± 120.24	
University or above	6,925 (51.83%)	496.75 ± 130.62		170 (1.52%)	385.79 ± 136.68	
**Smoking**			0.772			<0.001
Never	7,329 (54.81%)	477.61 ± 133.30		6,838 (61.10%)	276.24 ± 107.88	
Smoking cessation	3,007 (22.49%)	477.32 ± 138.22		906 (8.10%)	330.21 ± 137.38	
Smoking at present	3,035 (22.70%)	475.64 ± 136.54		3,447 (30.80%)	336.32 ± 129.67	
**Drinking**			<0.001			/
Yes	2,098 (19.39%)	440.70 ± 137.39		**/**	**/**	
No	8,723 (80.61%)	497.90 ± 131.68		**/**	**/**	
**Drinking**			/			<0.001
Never	/	/		6,617 (59.12%)	277.39 ± 108.66	
Drinking cessation	/	/		884 (7.90%)	296.69 ± 125.63	
Drinking at present	/	/		3,691 (32.98%)	338.62 ± 130.54	
**Hypertension**			<0.001			<0.001
Yes	5,020 (37.54%)	442.07 ± 138.50		2,562 (23.01%)	287.45 ± 118.45	
No	8,351 (62.46%)	498.31 ± 128.56		8,570 (76.99%)	302.54 ± 121.47	
**Hyperlipidemia**			<0.001			0.001
Yes	8,221 (79.26%)	473.32 ± 134.00		942 (8.59%)	312.18 ± 128.29	
No	2,151 (20.74%)	486.03 ± 132.88		10,025 (91.41%)	298.06 ± 120.24	
**Cardiovascular disease**			<0.001			<0.001
Yes	755 (5.66%)	408.03 ± 137.45		1,237 (11.10%)	267.43 ± 114.15	
No	12,576 (94.34%)	481.53 ± 133.82		9,908 (88.90%)	303.12 ± 121.19	
**Stroke**			<0.001			/
Yes	302 (2.25%)	384.48 ± 131.76		/	/	
No	13,069 (97.75%)	479.34 ± 134.48		/	/	

a Mean ± SD was used to indicate the age of the participants, and all other covariates are expressed as *n* (%).

b *p* values were derived from analysis of analysis of variance.

### 3.2 Associations between PEF and stroke in observational studies

**Figure 2:**
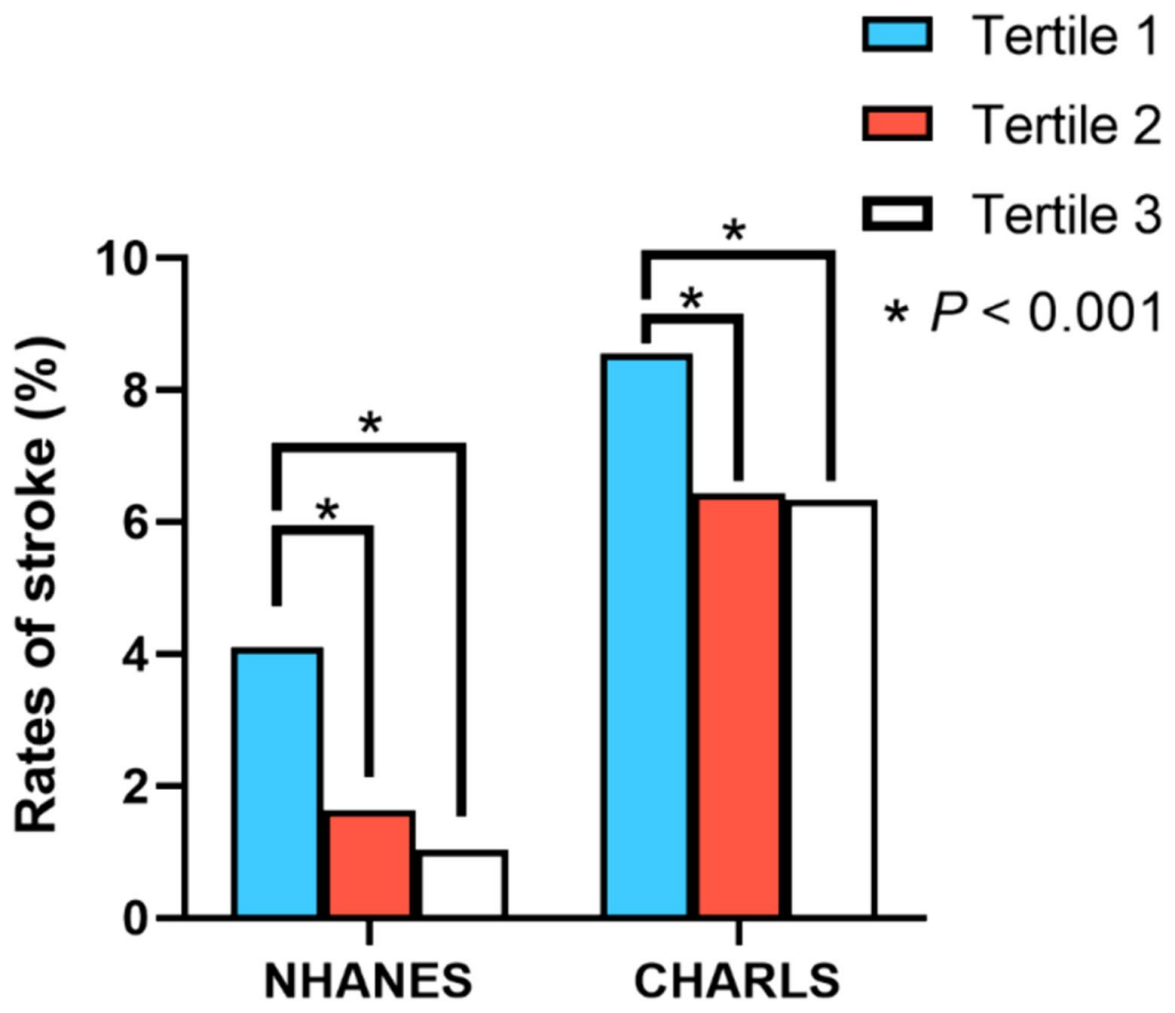
Differences in stroke prevalence and incidence rates among individuals with different PEF levels. *, *p* < 0.001 for univariate logistic regression and univariate Cox proportional hazards regression.

Based on the findings from both cross-sectional and cohort studies, participants within the second and third tertiles of PEF exhibited significantly reduced stroke prevalence and incidence relative to those in the lowest tertile, as depicted in Figure 2. In the cross-sectional analysis, outlined in Table 2, after adjusting for relevant covariates, individuals in the second (OR = 0.725, 95%CI = 0.530–0.991, *p* = 0.043) and third tertiles (OR = 0.623, 95%CI = 0.413–0.941, *p* = 0.024) demonstrated a significantly lower risk of stroke compared to the first tertile. Similarly, the longitudinal study results, presented in Table 3, indicated that individuals in the second (HR = 0.810, 95%CI = 0.684–0.960, *p* = 0.015) and third tertiles (HR = 0.786, 95%CI = 0.647–0.956, *p* = 0.016) of PEF levels had a lower incidence of stroke, with the risk diminishing progressively with higher PEF levels (*P*-trend = 0.009). Furthermore, restricted cubic splines revealed no evidence of a nonlinear relationship between PEF levels and stroke risk (Supplementary Figures S1, S2).

**Table 2:** Cross-sectional association between PEF and stroke.

Subgroup	PEF (L/min)	Prevalence	Model 1^a^			Model 2^b^			Model 3^c^		
			*p*	OR	95%CI	*p*	OR	95%CI	*p*	OR	95%CI
Tertile 1	332.48 ± 65.39	4.10%		Ref.			Ref.			Ref.	
Tertile 2	470.41 ± 33.49	1.64%	0.001	0.600	0.447–0.804	0.011	0.671	0.494–0.912	0.043	0.725	0.530–0.991
Tertile 3	628.76 ± 73.75	1.03%	<0.001	0.424	0.285–0.630	0.001	0.507	0.339–0.759	0.024	0.623	0.413–0.941
*P*-trend				<0.001			<0.001			*0.014*	

a Model 1: Adjusted for age, gender, BMI, race, marriage status, and education level.

b Adjusted Model 2: Additionally adjusted smoking status and drinking status.

c Adjusted Model 3: Additionally adjusted hypertension, hyperlipidemia, and cardiovascular disease.

**Table 3:** Longitudinal association between PEF and stroke.

Subgroup	PEF (L/min)	Incidence	Model 1^a^			Model 2^b^			Model 3^c^		
			*p*	HR	95%CI	*p*	HR	95%CI	*p*	HR	95%CI
Tertile 1	175.53 ± 45.18	8.55%		Ref.			Ref.			Ref.	
Tertile 2	300.32 ± 32.42	6.44%	0.012	0.805	0.680–0.954	0.015	0.811	0.685–0.960	0.015	0.810	0.684–0.960
Tertile 3	444.77 ± 74.54	6.34%	0.010	0.775	0.638–0.941	0.015	0.785	0.646–0.954	0.016	0.786	0.647–0.956
*P*-trend				*0.006*			*0.009*			*0.009*	

a Model 1: Adjusted for age, gender, BMI, place of residence, marriage status, and education level.

b Adjusted Model 2: Additionally adjusted smoking status and drinking status.

c Adjusted Model 3: Additionally adjusted hypertension, hyperlipidemia, and cardiovascular disease.

### 3.3 The association between PEF and stroke according to Mendelian randomization

In our study, we extracted 81 suitable genetic variants from the exposure dataset, the *F*-statistics of which ranged from 29.9 to 282.1. This range indicates that our instrumental variables are not at risk of being weak instruments. Detailed information about these genetic variants can be found in Supplementary Tables S4, S5.

Our two-sample MR analysis suggested a potential causal relationship between PEF and stroke. The main analysis method, the IVW approach, revealed that an increase in PEF was associated with a decreased risk of stroke (OR = 0.852, *p* = 0.046, 95%CI 0.727–0.997, Table 4). The results of the weighted median method and MR-Egger approach corroborate the directional consistency of the IVW findings.

**Table 4:** MR analysis between PEF and stroke.

Exposure	Outcome	Method	β	SE	*p*	OR	95%CI	Cochran’s Q (Q_*P*val, IVW)	MR-Egger (*P*val)	MR-PRESSSO (*P*val)
PEF	Stroke	MR Egger	−0.319	0.307	0.303	0.727	0.398–1.328	0.072	0.596	0.062
PEF	Stroke	Weighted median	−0.115	0.116	0.321	0.891	0.710–1.119			
PEF	Stroke	IVW	−0.161	0.081	0.046	0.852	0.727–0.997			
PEF	Stroke	Simple mode	−0.519	0.288	0.076	0.595	0.338–1.047			
PEF	Stroke	Weighted mode	−0.083	0.204	0.687	0.921	0.617–1.374			

### 3.4 Sensitivity analysis

We conducted several sensitivity analyses to evaluate the robustness of our results. In the observational study, we further investigated the association between stroke and every standard deviation increase in PEF (OR = 0.744, 95%CI = 0.636–0.870, *p* < 0.001 for cross-sectional analysis; HR = 0.891, 95%CI = 0.820–0.968, *p* = 0.006 for cohort analysis) and also analyzed the data before multiple imputation (Supplementary Tables S6, S7).

In the MR analysis, we found no evidence of influence from horizontal pleiotropy or heterogeneity (Table 4). Furthermore, bidirectional MR analysis supported the notion that reverse causation does not impact our findings (Supplementary Table S8). Leave-one-out analysis additionally confirmed that no single variant drove the results (Supplementary Figure S3). Scatter plots illustrating these findings are included in Supplementary Figure S4. The results of the sensitivity analysis support the robustness of the association.

## 4 Discussion

This study utilizes a cross-sectional survey from the United States to provide etiological clues for the association between low PEF and the risk of stroke. Furthermore, using a national cohort from China, it elucidates that individuals in the second and third tertiles of PEF function, compared to those in the first tertile, can reduce their risk by 19.0 and 21.4%, respectively. Additionally, MR analysis further confirms the causal relationship between the two. Several sensitivity analyses have reinforced the robustness of the results.

### 4.1 Comparison with other studies and mechanisms

Previous studies have emphasized lung function as a risk factor for cardiovascular diseases and highlighted the hazardous connection between the two conditions. Typically, these studies use patients with COPD to represent impaired lung function (8, 11). However, a restrictive lung pattern, a more common phenomenon closely associated with factors such as age and obesity and characterized by reduced lung volume and total lung capacity, even without airway obstruction, is also linked to an increased incidence of cardiovascular diseases (7). In recent epidemiological research, there has been a growing emphasis on understanding how lung function affects different subtypes of cardiovascular diseases, including heart failure, coronary heart disease, atrial fibrillation, and stroke (22–24). These studies commonly employ FEV1, FVC, or the FEV1/FVC ratio to assess lung function. PEF, as a potentially valuable lung function assessment indicator in the general population, is more suitable for community lung function monitoring and patient self-management (6). We used it as an indicator to evaluate lung function and further explored how limited lung function is a risk factor for stroke occurrence. Previous research has clarified that a dynamic trend of rapid lung function decline increases the risk of stroke occurrence (22). Another study has also demonstrated an association between lung function and fatal strokes (25). Similarly, an MR study supports our finding (24).

The underlying mechanisms by which declining lung function increases the risk of cardiovascular events remain unclear. Several possible explanations proposed by past studies are widely accepted, including changes in cardiac structure and compensatory capacity (7), pulmonary hypertension (8), inflammation, and oxidative stress (26). Due to changes in respiratory load, individuals with abnormal PEFs often experience significant changes in thoracic pressure, leading to considerable cardiac stress. Several scholars have noted that respiratory distress caused by airway constriction can lead to substantial changes in hemodynamics and cardiac structure (7, 8). The more severe the airflow obstruction is, the smaller the size of the left ventricle becomes, leading to reduced stroke volume and cardiac output (27). Such outcomes might stem from compromised ventricular filling, a consequence of hyperinflation, which can be observed even in the early stages of obstructive diseases (28). Therefore, changes in cardiac structure and hemodynamics may be potential mechanisms by which PEF influences stroke risk. A meta-analysis and case–control study indicated that pulmonary hypertension is an independent risk factor for stroke (29, 30). Strokes caused by limited lung function may be mediated by pulmonary hypertension. Pulmonary hypertension can increase right atrial pressure, potentially enabling paradoxical emboli to pass through a patent foramen ovale, thereby elevating the risk of stroke (31, 32). Systemic inflammation, a hallmark of cardiovascular diseases, contributes to their development and progression. Decreased lung function or resultant pulmonary hypertension correlates with endothelial dysfunction and increased levels of inflammatory mediators, such as C-reactive protein, interleukin-6, interleukin-8, and tumor necrosis factor-alpha (33–35). These elements stimulate and accelerate atherosclerotic plaque formation and are closely associated with cardiovascular events.

### 4.2 Implications

While identifying easily modifiable and reversible stroke risk factors is essential, many studies have focused on improving lung function through bronchodilator inhalation. However, the effectiveness of this treatment in enhancing lung function is limited and not substantial enough to reduce stroke risk. Moreover, while many studies concentrate on COPD patients, restrictive lung patterns resulting from subclinical lung dysfunctions, such as emphysema, are more prevalent in the general population (7). Due to the close link between limited lung function and cardiovascular diseases, treatment for one condition may affect the other (8). The clinical significance of this study lies in identifying limited lung function as an independent risk factor for stroke, which underscores the importance of close and convenient monitoring and multidisciplinary management of respiratory patients. Current guidelines also recommend continuous lung function tests for individuals at high risk of pulmonary diseases (36).

Stroke has become a major cause of death and disability in China, posing a significant threat to citizens’ health as a major chronic noncommunicable disease. In 2020, 17.8 million adults in China were estimated to experience stroke, and 2.3 million of these patients died as a result. The estimated cost of hospitalization for stroke in 2020 was 58 billion yuan, with patients paying approximately 19.8 billion yuan (37). Even though the incidence of stroke has been decreasing over the years in developed countries, the annual costs per stroke patient in the United States and the United Kingdom have reached $59,900 and £45,409, respectively (38, 39). This study utilized a national cross-sectional survey from the U.S., a national cohort study from China, and a large-sample MR study in a European population. It presented progressively strengthening evidence of the association between lung function and stroke risk, offering insights from diverse ethnic populations. The escalating global burden of stroke strongly indicates that current primary prevention strategies for stroke and cardiovascular diseases are either not widely implemented or insufficiently effective. This study introduces a lung function monitoring approach for community and personal care, targeting stroke’s primary prevention policies (40).

### 4.3 Limitations

In our observational study, despite controlling for sociodemographic characteristics and other significant covariates, some potential unknown confounding factors inevitably remained unadjusted. Even after sensitivity analyses in MR have addressed the potential impacts of horizontal pleiotropy and heterogeneity, inherent confounding biases may still be introduced into the results owing to inherent flaws in phenotype definitions within GWAS data, such as unclear phenotype definitions. Moreover, although stroke assessment is based on the judgment of doctors and experts, reliance on self-report questionnaires can still introduce reporting bias. Furthermore, the stroke patients suitable for personal questionnaire surveys in our study had nonfatal strokes. Caution should be exercised in extrapolating the findings to the association between lung function and fatal strokes. Additionally, our study did not distinguish between various types of stroke, such as ischemic or hemorrhagic stroke, but these distinctions did not affect the internal consistency of the research. Finally, while our study provides strong evidence supporting a causal relationship between declining lung function and stroke, further mechanistic research is needed to explore the underlying mechanisms of this association.

## 5 Conclusion

In summary, this study, using data from diverse ethnic populations, revealed that impaired lung function is a risk factor for stroke. Stroke poses a significant public health challenge in both developing and developed countries, and stroke-related pulmonary diseases are a substantial economic burden on society and patients. By utilizing PEF, an easily measurable indicator of lung function, this study offers potential for respiratory disease patients and the general population to prevent stroke through self-monitoring of lung function.

## Data availability statement

Publicly available datasets were analyzed in this study. This data can be found here: CHARLS: https://charls.pku.edu.cn/; NHANES: https://www.cdc.gov/nchs/nhanes/; PEF GWAS data: https://www.ukbiobank.ac.uk/; Stroke GWAS data: https://pubmed.ncbi.nlm.nih.gov/29531354/, and they are publicly available.

## Ethics statement

The studies involving humans were approved by the Biomedical Ethics Review Broad of Peking University (IRB00001052-11015). The studies were conducted in accordance with the local legislation and institutional requirements. Written informed consent for participation was not required from the participants or the participants’ legal guardians/next of kin in accordance with the national legislation and institutional requirements.

## Author contributions

JW: Writing – original draft, Software, Methodology, Investigation, Formal analysis, Data curation, Conceptualization. JL: Writing – review & editing, Writing – original draft, Validation, Software, Resources, Methodology, Investigation, Formal analysis, Data curation, Conceptualization. YZhe: Formal analysis, Data curation, Writing- review & editing, Software. MH: Writing – original draft, Software, Methodology, Investigation, Formal analysis. KW: Writing – original draft, Software, Methodology, Formal analysis. KL: Writing – original draft, Software, Methodology, Formal analysis. YZha: Writing – original draft, Software, Methodology, Formal analysis. WZ: Writing – review & editing, Supervision, Software, Methodology, Investigation, Formal analysis. RC: Writing – review & editing, Visualization, Validation, Supervision, Software, Resources, Project administration, Methodology, Investigation, Funding acquisition, Formal analysis. FL: Writing – review & editing, Visualization, Validation, Supervision, Project administration.

## Footnotes

^1^ www.medsta.cn/software
